# The Core–Shell Conformational Space of Compartmentalized Single‐Chain Nanoparticles by Paramagnetic and Hyperpolarized NMR Spectroscopy

**DOI:** 10.1002/advs.202510909

**Published:** 2025-11-30

**Authors:** Federico Faglia, Justus F. Thümmler, Christopher Pötzl, Milan Zachrdla, Ertan Turhan, Dennis Kurzbach, Wolfgang H. Binder

**Affiliations:** ^1^ Faculty of Chemistry Institute of Biological Chemistry University of Vienna Währinger Str. 38 Vienna 1090 Austria; ^2^ Institute of Chemistry Faculty of Natural Science II (Chemistry, Physics and Mathematics) Martin Luther University Halle‐Wittenberg Von‐Danckelmann‐Platz 4 D‐06120 Halle (Saale) Germany; ^3^ Doctoral School of Chemistry Faculty of Chemistry University of Vienna Währinger Str. 38 Vienna 1090 Austria

**Keywords:** atomistic structure determination, hyperpolarization, molecular dynamics simulations, NMR, single‐chain nanoparticles

## Abstract

Single‐chain nanoparticles (SCNPs) are formed by the collapse of individual polymer chains, generating entities comparable to proteins in size, internal structure, and function. Especially, the formation of hierarchies induced by complex folds of linear polymer chains can result in internalized compartments, reminiscent of pockets in enzymes. However, direct experimental access to their architecture or mode of contact remains a challenge. Here, the conformational organization of a prototypical amphiphilic SCNP is dissected to reveal conformational details of its internally heterogeneous morphology driven by site‐specific intramolecular compaction. Using a synergistic combination of unconventional paramagnetic NMR, hyperpolarized water‐based dissolution dynamic nuclear polarization (d‐DNP), and NMR‐guided molecular dynamics simulations, intramolecular structures and solvent accessibility are mapped at atomistic resolution. These findings uncover distinct nanoscopic compartments formed via back‐folding of PEG side chains toward the SCNP backbone. Furthermore, these compartments shield internal segments, mimicking hydrophobic pockets found in folded proteins. Thus, this work introduces a transferable methodology for probing functional compartmentalization in synthetic macromolecules. It provides a tool for rationally designing next‐generation nanomaterials and enzyme mimetics with programmable internal order via residue‐resolved structural information. At the same time, this method's prowess is evidenced through a high‐resolution description of the local conformations found within hierarchically structured SCNPs.

## Graphical abstract

Combinations of integrative NMR spectroscopy and molecular dynamics simulations reveal the internal structural dynamics of single‐chain nanoparticles.

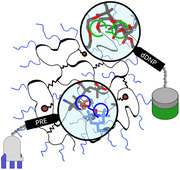

## 1 Introduction

Single‐chain nanoparticles (SCNPs) are an emergent class of soft nanomaterials formed via intramolecular collapse of singular, linear polymers.^[^ ^1^, ^2^, ^3^, ^4^, ^5^, ^6^, ^7^ ^]^ These synthetic entities typically span 5 to 20 nm in hydrodynamic radius, corresponding to the dimensions of a collapsed “polymer globule”.^[^ ^8^ ^]^ What distinguishes them is a conceptual analogy to folded proteins, comparable in size, compartmentalization, and catalytic function. Indeed, SCNPs feature discrete nanocompartments within a single macromolecule reminiscent of a protein's hydrophobic core.^[^ ^1^, ^2^ ^]^ Such compartmentalization enables the creation of localized environments, thereby not only imitating biological nanoscale organization but also expanding the functional landscape of polymer‐based systems for catalysis,^[^ ^9^, ^10^ ^]^ sensing,^[^ ^11^, ^12^ ^]^ drug delivery,^[^ ^13^, ^14^ ^]^ and biomimetic applications.^[^ ^15^, ^16^ ^]^ In contrast to folded proteins, however, the polymers are often covalently crosslinked (between 3 and >20 crosslinking sites/chain), thus fixing the polymer's fold after the initial chain collapse. These internal compartments arise from a sophisticated orchestration of chemical and topological design: copolymer composition, amphiphilic interactions, the conditions of collapse (solvent/temperature or thermal transitions of polymer segments), or stimuli‐responsive cross‐linking modalities that guide the folding trajectory toward structured nanoarchitectures. The resulting SCNPs may adopt core–shell morphologies or multichambered organizations, wherein microenvironments of varying polarity and rigidity can be tailored to specific tasks such as dye encapsulation or site‐specific catalysis.^[^ ^6^ ^]^

Describing the molecular heterogeneities formed during chain collapse within SCNPs using analytical methods is often challenging, due to the precision and accuracy of standard measurement procedures such as in size‐exclusion chromatography, dynamic light scattering, or diffusion‐ordered nuclear magnetic resonance (NMR) spectroscopy (DOSY).^[^ ^8^, ^17^ ^]^ Their small size and the polymers' inherent multiscale dynamics, prevailing despite the covalent crosslinking, demand an integrative approach covering multiple high‐resolution techniques (see, e.g., ref. [2] and references cited therein).

Recent advancements in solution‐state techniques, most notably optoelectronic labeling (Förster resonance transfer^[^ ^18^ ^]^ or fluorescence‐lifetime spectroscopy)^[^ ^19^ ^]^, DOSY^[^ ^20^ ^]^, Overhauser dynamic nuclear polarization (ODNP)^[^ ^21^ ^]^, continuous wave‐electron paramagnetic resonance (CW‐EPR)^[^ ^20^, ^22^ ^]^, and fluorescence correlation spectroscopy^[^ ^20^, ^23^ ^]^ have been employed to study SCNPs in their native solution states. DOSY‐NMR, in particular, provides sensitive insight into hydrodynamic size changes accompanying single‐chain collapse, while CW‐EPR yields compactness estimates of intra‐SCNP domains.

Despite such a broad methodological portfolio, only indirect compartment mapping has been reported so far. The structural information obtained thereby neither provides a resolved structure nor an interaction profile for specific polymer segments, as is often reported, e.g., for intrinsically disordered proteins by mapping specific amino‐acid interactions.^[^ ^24^, ^25^, ^26^ ^]^As a result, despite their conceptual elegance, a direct structural elucidation of SCNPs at high resolution has not yet been achieved.

It would be desirable to usher in a paradigm wherein soft matter engineering of SCNPs converges with the atomistically detailed conformational analyses typical of structural biology. SCNPs would transition from notional constructs to functionally characterized nanoplatforms. Indeed, their tunable architectures, if structurally resolved, would enable precise control over internal compartmentalization.

To foster this transition, we herein present a detailed structural dissection of compartmentalized SCNPs, revealing a bimodal morphology that emerges from covalent intramolecular folding. By integrating tailored NMR techniques with hyperpolarization strategies^[^ ^27^, ^28^ ^]^ and NMR‐validated molecular dynamics (MD) simulations, we directly observe how distinct nanoscopic domains are structured and how solvent accessibility is partitioned within a single macromolecule. This work, thus,

- constitutes an innovative methodological foundation for deciphering this class of precision‐engineered soft materials whose internal structure can be programmed to control molecular function at the nanoscale.
- provides the first high‐resolution description of local compartments within SCNP reminiscent of hydrophobic pockets within globular proteins.

## 2 Results and Discussion

**Figure 1:**
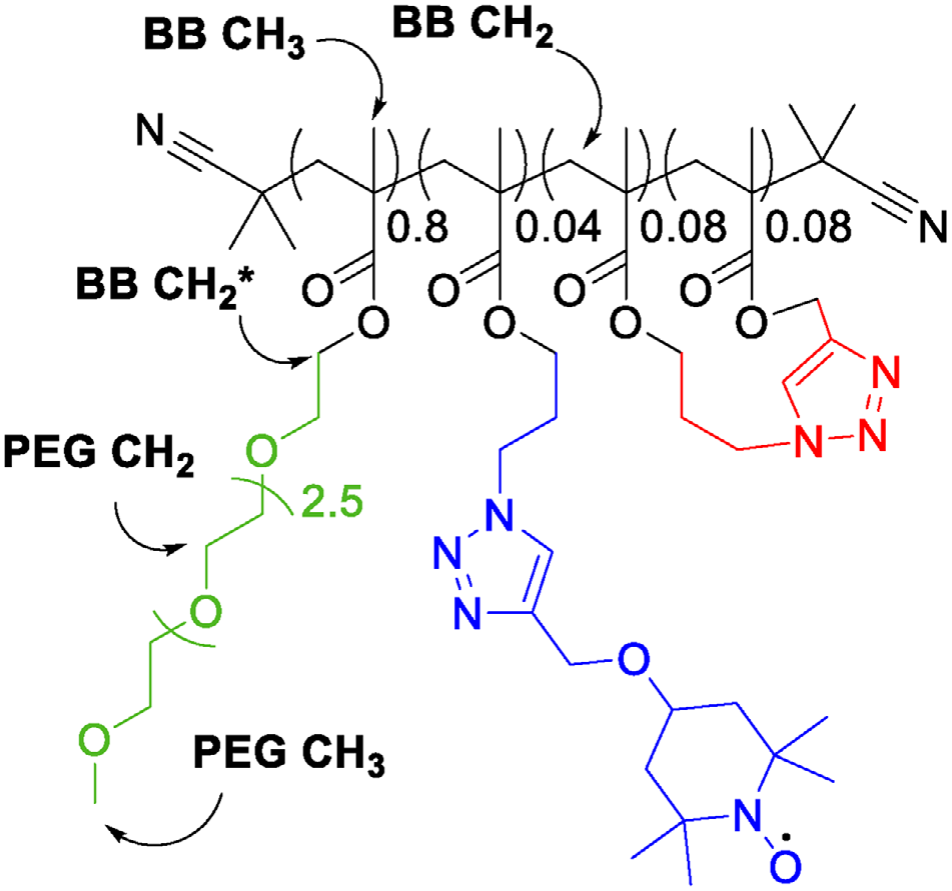
Chemical structure of the SCNP under study. Statistically distributed crosslinking sites (red) cause the local collapse of the polymer chain (black). PEG (green) side chains stabilize the SCNP in aqueous environments, and a TEMPO label (blue) serves as a local structural probe. The coarse nomenclature used herein is indicated (BB: “back bone”).

The linear polymer underlying the SCNPs investigated in this study was synthesized via RAFT polymerization of methacrylate monomers with subsequent end‐group removal, as reported in earlier studies (*M_n_* = 36.1 kDa; *M_w_/M_n_* = 1.7; degree of polymerization = 129).^[^ ^23^ ^]^ The methacrylate units were functionalized with polyethylene glycol (PEG) side chains (80 mol% of the monomer), azides (12 mol%), and alkynes (8 mol%; also indicated in **Figure** **1**). Single‐chain collapse and crosslinking were achieved by an aqueous copper‐catalyzed azide‐alkyne cycloaddition (CuAAC). Subsequently, the excess azide moieties were further functionalized using alkyne‐modified TEMPO (2,2,6,6‐Tetramethylpiperidinyloxyl) in a one‐pot approach. The resulting SCNPs display a hydrodynamic radius of 5.2 nm, corresponding to a partially collapsed globule‐like state (see Figures S1–S6, Supporting Information).^[^ ^23^ ^]^ Further analytical data can be found in the Supporting Information and ref. [23].

For the NMR experiments described here, the SCNPs were dissolved in D_2_O mixtures through stirring and subsequent sonication. The final sample concentration was 2 mg mL^−1^ (all details can be found in the Experimental Section in the Supporting Information).

The three distinct functional moieties synergistically guide the SCNP self‐folding and internal compartmentalization. The PEG chains (Figure 1, green) contribute to stabilizing water interactions of the collapsed globules, allowing intramolecular folding in aqueous environments without precipitation.

The next central design element of the polymer is the crosslinking unit (Figure 1, red), which induces covalent loop formation. This crosslinking results in localized, nanoscale compartments isolated from the bulk solvent, i.e., microdomains based on local chain collapse within the SCNP.

Finally, the labeling site (Figure 1, blue) enables post‐polymerization attachment of sensors or labels. Such labels are particularly valuable for probing an SCNP's internal microenvironment by site‐selective spectroscopic methods.

Herein, to probe the local organization of these compartments, we attached a stable radical (TEMPO) spin label (SL) to explore the nanoscopic environments of the SCNPs. We employ the SL as a paramagnetic relaxation enhancer to resolve segmental compaction through distance‐dependent interactions relative to the labeling site.

In the following, we first describe conventional NMR experiments that reveal the core–shell architecture of the probed SCNP, followed by the unconventional SL‐based paramagnetic relaxation enhancement (PRE) and dissolution dynamic nuclear polarization (dDNP) experiments to describe local structures of the different SCNP domains.

### 2.1 The Dual SCNP Composition

**Figure 2:**
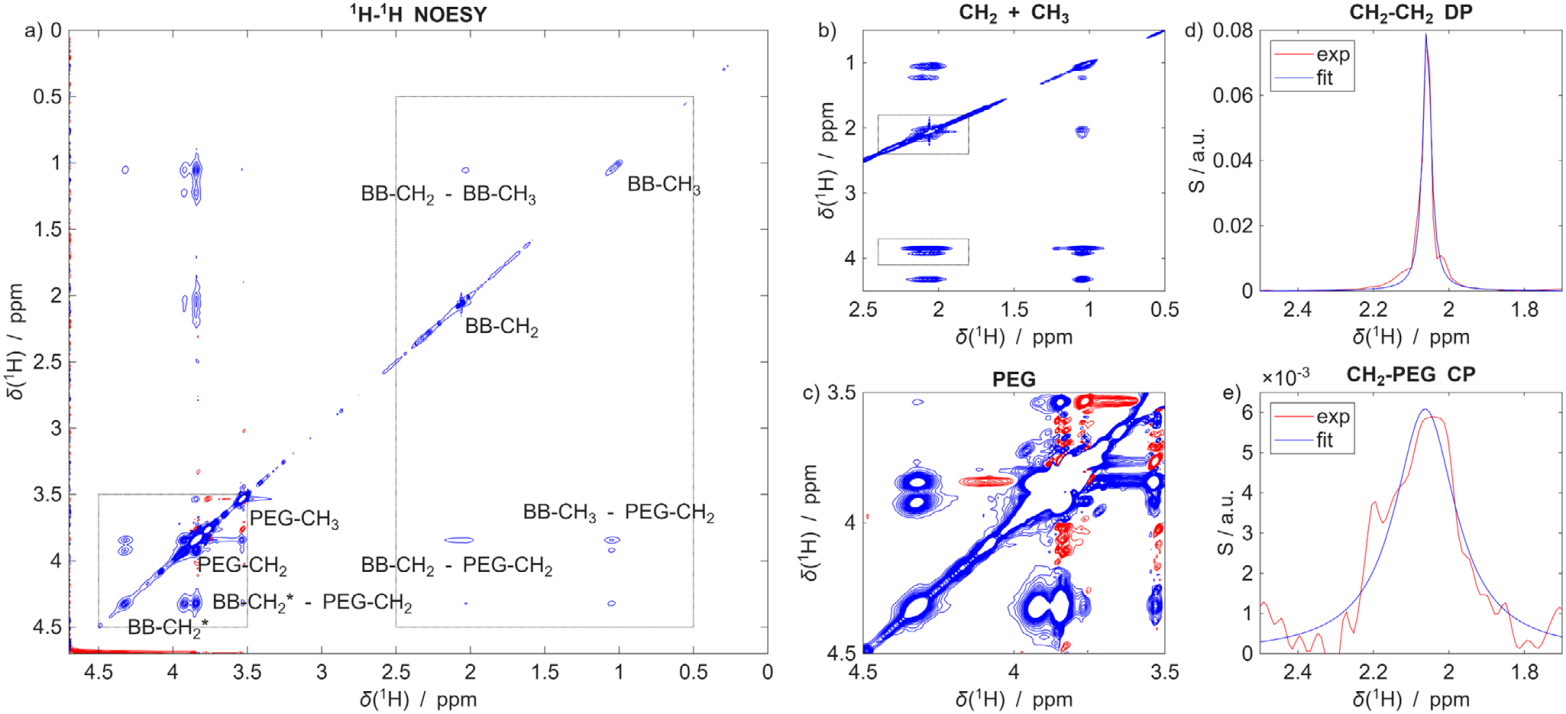
a) Proton‐only NOESY spectrum of the SCNP recorded with a mixing time of 1 s. The resonance assignment is indicated. b, c) Zoom‐ins of regions highlighted with the squared boxes in panel (a). Note that the contour levels are chosen differently to visualize weaker signals in the zooms. d, e) Projections of the CH_2_‐PEG cross peaks (CP) boxed in panel (b) and of the corresponding diagonal peak (DP). The blue solid line corresponds to a Lorentzian fit. Evidently, the DP consists of two components superimposed, while the CP reflects only the broad component.

Our initial spectroscopic characterization of the SCNP system was conducted using conventional ^1^H‐^1^H NOESY (nuclear Overhauser enhanced spectroscopy) experiments (**Figure** **2**). In accordance with previous reports on compartmentalized SCNPs,^[^ ^20^ ^]^ spectra exhibited features consistent with the structure shown in Figure 1. Note that only a coarse resonance assignment is indicated for the sake of simplicity (cf. Figure 1; full assignment in ref. [20] and the Table S1 and Figure S7, Supporting Information).

Upon closer inspection, an important detail emerges: in the NOESY spectra (Figure 2), the methyl and methylene group signals consistently display two distinct spectral components. Specifically, one observes a sharp, well‐resolved signal superimposed with a considerably broadened component—a feature observed consistently in NMR of SCNP^[^ ^2^, ^23^, ^29^, ^30^ ^]^, but which has gone unnoticed so far. Figure 2b shows a zoom onto the backbone (BB) CH_2_ and CH_3_ region of the NOESY spectrum between 0.5 and 2.5 ppm and onto the BB‐PEG cross peaks resonances around 4 ppm (Figure 2c highlights the PEG region for additional clarity).

The peak duplication is best visualized through the projections in Figure 2d,e of the BB‐CH_2_‐to‐PEG‐CH_2_ cross peaks (CP) and the CH_2_‐CH_2_ diagonal peak (DP), marked by the boxes in Figure 2b. The DP is dominated by a sharp resonance with a line width of Γ = 0.03 ± 0.01 ppm (derived from the blue fit), superimposed on a broad line appearing at the signals’ flanks. In contrast, the cross peak shows a much broader line width of Γ = 0.20 ± 0.01 ppm and no contribution of any sharp resonance. Similar observations were made for all other CH_2_ and CH_3_ resonances as well as the sidechain terminal, PEG‐CH_3_‐to‐BB‐CH_2_ CP (all assignments and zooms on all cross‐peaks in Figures S7–S9, Supporting Information).

HSQC spectra (Figure S10, Supporting Information) similarly showed this duality, manifesting again as sharp and broad resonances superposing for all CH_2_ and CH_3_ signals.

Considering that the chemical structure (Figure 1) would only give rise to two backbone CH_3_ and one CH_2_ resonance, the observation of peak duplication strongly suggests at least two co‐existing conformational states. Further, taking into account the dependence of the NMR line width Γ on the local conformational mobility (where slower tumbling causes broader lines)^[^ ^31^ ^]^, these data point toward one resonance corresponding to mobile, solvent‐exposed segments (likely in an extended conformation), leading to the sharp resonances, and another indicative of restricted, immobilized regions (presumably buried within compacted SCNP compartments) leading to the broad resonance. Notably, only the broadened components show NOE cross‐peaks, i.e., through‐space spatial proximity (cf. Figure S11, Supporting Information), between the BB‐CH_2_ and BB‐CH_3_ groups to the PEG sidechains. These resonances, thus, indicate:

- that the PEG chains fold back to sample the methacrylate backbone.
- population of a compacted state with reduced mobility underlying BB‐PEG contacts.

These observations motivated our subsequent experiments, as they pointed toward a direct handle to selectively probe and distinguish between compacted and extended regions of the SCNP at the level of individual chemical groups.

### 2.2 The Structure of Compacted Segments

Next, we performed paramagnetic relaxation enhancement (PRE) experiments^[^ ^31^, ^32^ ^]^, capitalizing on the TEMPO SL. The aim was to gain residue‐resolved insights into label proximity across the polymer structure. The experiment is based on the concept that the closer a proton approaches the unpaired electron of the SL, the faster its nuclear spin relaxation.^[^ ^32^ ^]^ Particularly, the transverse relaxation rate *R*_2_ is affected most strongly, thereby exhibiting a steep *R*_2_ ∝ *r* ^−6^ dependence on the SL‐to‐proton distance *r*. PREs are a powerful tool widely used in the context of structural biology.^[^ ^33^, ^34^, ^35^, ^36^, ^37^, ^38^, ^39^ ^]^ Herein, we expand their use to polymers in suspension and SCNP.

**Figure 3:**
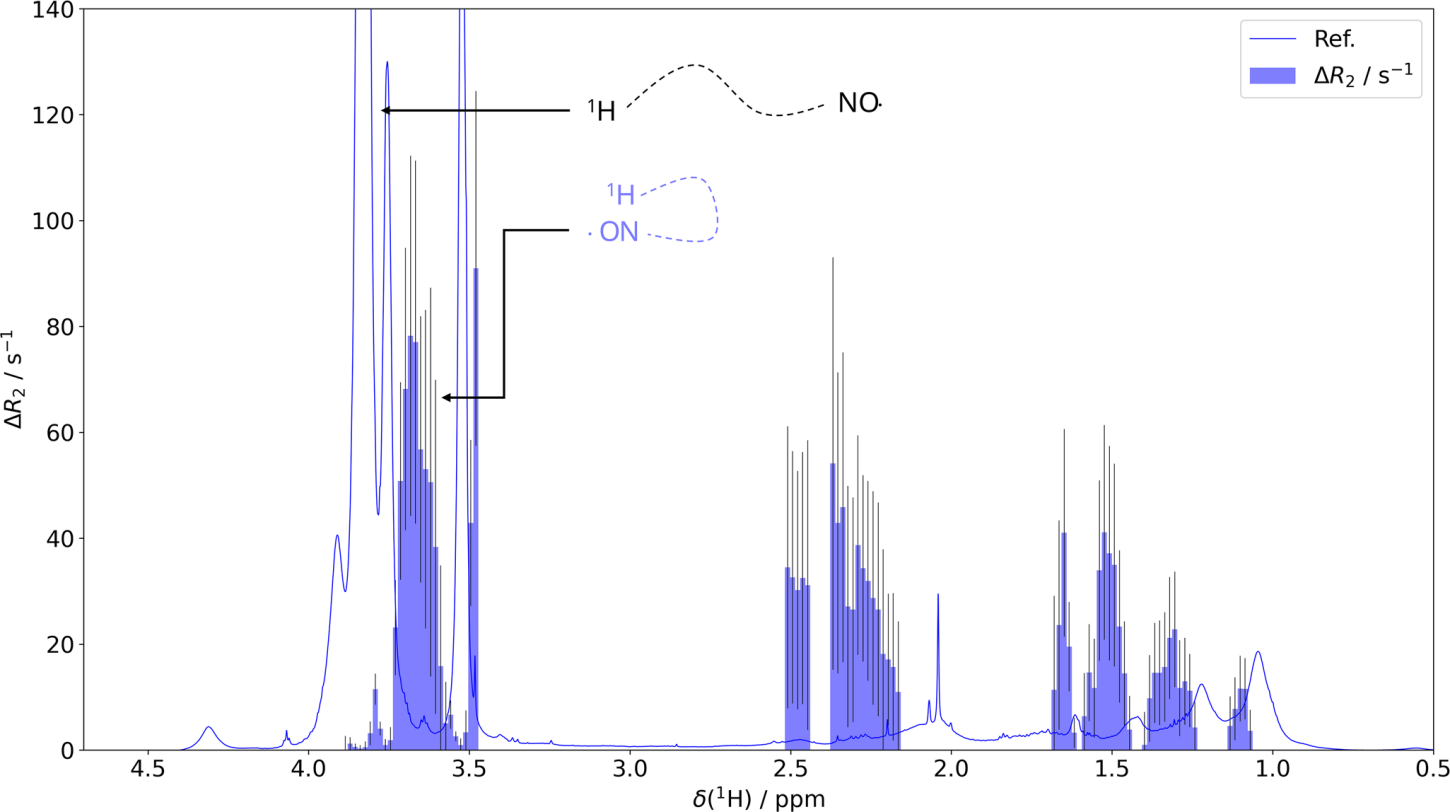
Paramagnetic relaxation enhancement PRE, results are represented as the change in transverse proton relaxation rates ΔR_2_ (blue bins) upon TEMPO reduction in dependence on the ^1^H chemical shift. The proton spectrum of our SCNP is superimposed as a guide to the eye. The inset highlights that resonances from protons proximate to the SL display high Δ*R*_2_ values, while distal SCNP protons lead to minor or negligible values.

The data shown in **Figure** **3** represent the changes in transverse relaxation rates (Δ*R*_2_) between the paramagnetic (active SL; N─O⋅) and diamagnetic (SL reduced with 2 eq. ascorbate; N─OH) states, *ceteris paribus* (all experimental details can be found in the Experimental Section). These values are superimposed onto the conventional ^1^H NMR spectrum. In brief, since PREs scale with *r* ^−6^, higher Δ*R*_2_ values (blue bins in Figure 3) indicate closer proximity between the paramagnetic tag and the proton underlying an affected resonance.

Notably, we could not observe any significant PRE for any of the sharp, strong peaks of the ^1^H spectrum. In other words, the *R*_2_ rate constants were identical for both states (Figure S12, Supporting Information). In contrast, PREs were only pronounced at the resonances’ broadened shoulders. This was observed consistently for all CH_3_, CH_2,_ and PEG signals. Considering Δ*R*_2_ ∝ *r* ^−6^, this observation confirms that the paramagnetic label is not in contact with mobile, extended conformations that give rise to sharp resonances.^[^ ^35^, ^40^ ^]^ Instead, it localizes in proximity to the structurally compacted components underlying broadened signals. In other words, the PRE experiments show that the SL is embedded in the collapsed pockets identified upstream by NOESY and HSQC.

Importantly, the pattern of broad and sharp resonances superposing remained even when the SL was replaced with a diamagnetic tag or reduced into the hydroxy amine (see Figures S13–S18, Supporting Information), showing that the line broadening is not merely due to proximate labels but indeed due to locally reduced mobility.

Moreover, it should be noted that also for the PEG, significant PRE effects were observed only on the low‐field flanks of the main resonance (≈3.5–4 ppm). In contrast, the majority of the side chain resonances remained unaffected. This pattern indicates that a large share of the PEG chains remain solvent‐exposed, thus corroborating a picture of an internally differentiated SCNP architecture: mobility‐restricted, compartmentalized segments as a result of PEG back folding co‐exist with more flexible, solvent‐exposed domains.

In our earlier studies, the presence of hydrophobic compartments has been investigated by co‐solubilizing fluorescent tracers such as pyrene or Nile red, which were designed to exhibit differential enrichment between compartments.^[^ ^20^, ^23^ ^]^ However, in contrast to the methodology developed herein, no residue‐specific information could be obtained due to the resolution constraints of such methods. Besides, the selectivity of such tracers often remained low. Thus, the herein‐proposed NMR methodology represents a significant step forward toward atomistically detailed, high‐resolution SCNP structure determination, enabling the understanding of contacts between polymer segments.

### 2.3 The Structure of Extended Segments

To further dissect the spatial arrangement and solvent accessibility of the SCNP components, we employed the dissolution dynamic nuclear polarization (d‐DNP) technique^[^ ^41^ ^]^ using hyperpolarized water (HyperW). This approach allows for mapping solvent‐exposed sites at high sensitivity. It was recently established for proteins and nucleic acids^[^ ^42^, ^43^, ^44^, ^45^, ^46^, ^47^, ^48^, ^49^, ^50^, ^51^, ^52^, ^53^, ^54^, ^55^, ^56^, ^57^, ^58^, ^59^, ^60^ ^]^ and is herein extended to synthetic polymers.

**Figure 4:**
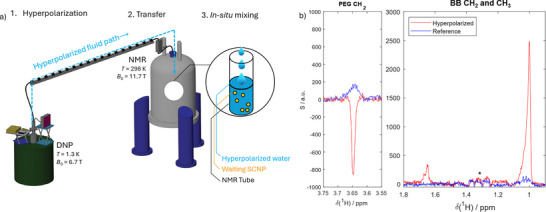
a) Sketch of the devised dDNP experiment 1.) Water is hyperpolarized by DNP using cryogenic dynamic nuclear polarization (DNP). 2.) Upon rapid dissolution, the hyperpolarized water is transferred to the NMR spectrometer for detection. 3.) The hyperpolarized water (HyperW) is mixed in situ within the NMR spectrometer with an SCNP solution. After completion of the mixing event, fast spectra with over 100‐fold boosted ^1^H‐sensitivity for solvent‐exposed sites are recorded. b) Comparison of the hyperpolarized spectrum (red) with a conventional reference spectrum (blue) obtained with the same sample and experiment, but after decay of the hyperpolarization. While the former shows strong signal intensities for a subset of sharp resonances, the latter only led to weak signals. Notably, the PEG CH_2_ resonance is inverted under HyperW conditions. The resonances marked with the * stem from the used DNP matrix.

As shown in the workflow diagram in **Figure** **4**a, water supplemented with small amounts of radicals as polarization agents (PA) is treated in a dedicated DNP apparatus at cryogenic temperatures (here, o*T*_DNP_ = 1.3 K, *B*_0, DNP_ = 6.7 T) with microwave irradiation, resulting in strong ^1^H signal enhancements.^[^ ^61^ ^]^ The water pellet is then dissolved in hot D_2_O and rapidly transferred to the NMR spectrometer. There, it is mixed with the SCNP solution in situ, i.e., directly within the NMR tube waiting in the spectrometer. This results in a transfer of signal enhancement from the hyperpolarized water to the solvent‐exposed protons of the SCNP through NOEs.^[^ ^58^, ^62^ ^]^

Thus, a transient but significant enhancement of NMR signal intensity is achieved selectively for solvent‐accessible sites (see ref. [59] for a comprehensive description of the entire experimental workflow).

In the resulting spectrum (Figure 4b), HyperW substantially enhances a subset of the ^1^H resonances. Several features must be considered:

- Concerning the SCNP backbone: Only the sharp methyl resonances of the BB CH_2_ and CH_3_ are enhanced by factors ε of 201 and 134, respectively (red spectrum), relative to the reference spectrum (blue spectrum, recorded with the same sample and experiment, but after complete decay of the hyperpolarization). In contrast, the broadened spectral components remained unaffected. This selective amplification of only the narrow signals provides evidence that the underlying protons reside in solvent‐exposed regions of the SCNP. In contrast, the absence of hyperpolarization transfer to the broad resonances further supports their assignment to protons sequestered within compacted, internal compartments, i.e., regions shielded from the solvent and thus inaccessible to HyperW.
- Concerning the PEG side chains: The PEG‐CH_2_ signals display a negative enhancement (signal inversion) by a factor of ≈−4 of the sharp main component of the ^1^H spectrum (again, the broad shoulders did not receive any hyperpolarization from the water). Our earlier work^[^ ^58^ ^]^ showed that such negative enhancements stem from direct NOE between HyperW and regions of the macromolecule with high conformational mobility – in other words, direct contact between mobile PEG side chains and the hydration water. Indeed, it is well documented that high segmental mobility entails a negative magnetization transfer from water to solute.^[^ ^63^, ^64^ ^]^ Hence, this observation directly points toward solvent exposure of highly mobile PEG side chains, as opposed to more restricted backbone motions entailing a positive NOE.

On the one hand, these findings validate the dual‐population interpretation (compacted and expanded, solvent‐exposed regions) derived from NOESY, HSQC, and PRE experiments by showing that only a subset of protons is solvent‐exposed for both BB and PEG. On the other hand, they establish dDNP as a tool for distinguishing solvent‐exposed from buried structural motifs in folded synthetic macromolecules. Besides, the complex HyperW approach is justified as conventional experiments such as NOESY (Figure 2) or water‐selective NOESY^[^ ^65^ ^]^ could not resolve the solvent exposure at the same precision due to prohibitively weak water‐SCNP cross peaks and low signal intensities of the broad spectral components.

**Figure 5:**
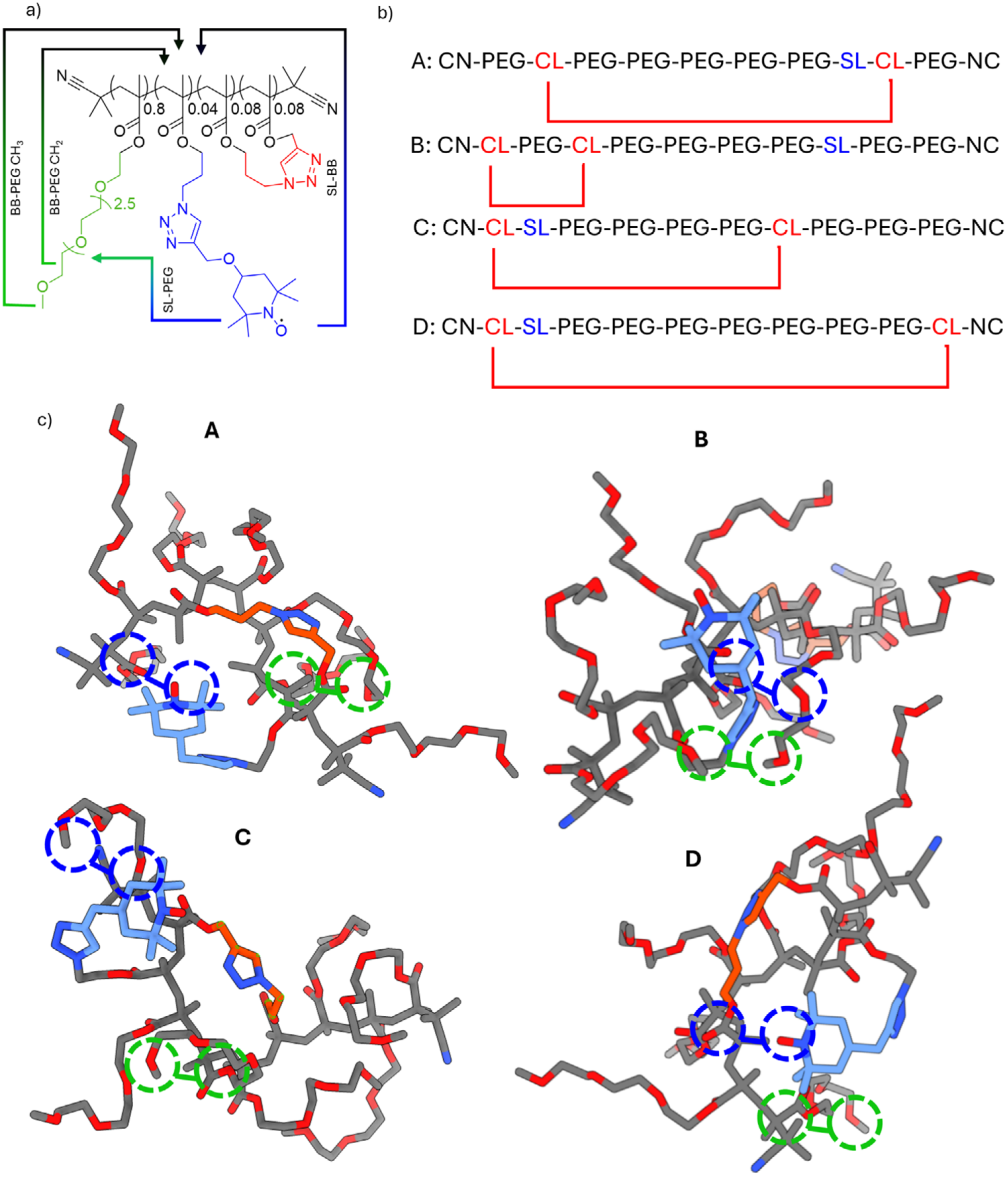
a) Visualization of the identified intrachain contacts for the compacted SCNP compartments. b) Examples of polymer configurations probed by MD simulations. ‘PEG’ denotes monomers with a PEG side‐chain, ‘SL’ the labeling site monomers, and ‘CL’ the cross‐linking residues. c) Energy‐minimized structures corresponding to the sequences shown in panel (b). All four simulations reproduced the NMR constraints, with the SL (blue) embedded compactly between adjacent PEG chains (blue circles). The position of the CL site (red) did not alter this configuration. The PEG chains’ terminal methyl groups also form contacts with the BB (green circles) in line with the experimental NOESY data. The encircled contacts are only a visualization of a representative subset of all formed contacts; for distance distributions, see the Supporting Information.

A schematic summary of our findings is presented in **Figure** **5**a, which illustrates the modular SCNP design and the identified contacts within the compacted compartments.

Finally, to underline these coarse‐grained structural constraints with an atomistically detailed picture of the local compartments, we performed all‐atom molecular dynamics (MD) simulations in explicit water (all details in the Experimental section). To approximate the statistical nature of the SCNP copolymers, we chose a viable minimal motif corresponding to the structure shown in Figure 1 (7:2:1, PEG, cross‐linking, and TEMPO sidechains, respectively) and randomly varied the positioning of the different residues within the decamer (see Figure 5b). For each variant, the starting structures were obtained by initial energy minimization in‐vacuo using MP2‐type molecular dynamics. These structures were then solvated and evolved by unconstrained classical MD using the CHARMM 36 force‐field until a stable plateau in terms of the RMSD from the starting configuration was reached (Figure S11a, Supporting Information). A representative set of the resulting configurations is shown in Figure 5c. In all simulations. We found that the SL folded back toward the BB and embedded in adjacent PEG chains to form a compacted segment. Distal PEG chains, in contrast, remained elongated, reaching further into the solvent. A statistical analysis of PEG‐SL and PEG‐BB distances (Figure S11b,c, Supporting Information) confirmed the relevance of the shown snapshots. The simulated models, thus, all reproduced the NMR‐derived contacts even without any structure constraints, which, vice versa, confirm these structures as plausible models for the SCNP pockets. It is important to note that this convergence of NMR constraints with MD results can be understood as an experimental vs. computational cross‐referencing. In other words, the MD simulations correctly predict the experimentally observed structural features.

It should be stressed that the presented minimal models reflect only a minute fraction of the possible local configurations within a full SCNP structure. Interpreting them should, thus, be done carefully. However, the convergence between experimental and computational data clearly shows that the presented models reflect possible instantiations of how the experimental data is reflected in a part of the SCNP conformational space. Hence, even if our models do not depict the entire SCNP, they nonetheless provide a visualization of how the NMR constraints can translate into polymer chain configurations.

Finally, we want to emphasize that the presented integrative methodology provides model‐free and atomistically resolved structural models of SCNPs. These features represent essential advantages over the often‐used scattering techniques (such as SAXS) when aiming to resolve local structural elements. Indeed, while scattering methods can provide structural constraints on length scales typical >1 nm, our NMR/MD‐based approach can provide complementary data on local structural arrangements at atomistic detail.

## 3 Conclusion

This study reveals the structural features of compacted compartments in amphiphilic SCNPs. We demonstrate that the PEG side chains fold back onto the hydrophobic backbone, forming localized domains in which dynamic and solvent‐shielded environments co‐exist. These compartments encapsulate paramagnetic labels, giving rise to a structurally and functionally differentiated internal organization. This finding goes beyond the previously reported information, now providing a first‐of‐its‐kind atomistically detailed description of domain‐specific encapsulation and the steric shielding by the PEG chains, which is central for future catalytic use of such domains.

Moreover, our findings highlight that SCNPs do not adopt homogeneous globular conformations but instead exhibit a heterogeneous spatial distribution of mobility and solvent‐accessibility, features reminiscent of biological macromolecules such as globular protein folds. The presence of buried compartments and solvent‐exposed domains underscores the potential of SCNPs to act as compartmentalized nanoreactors or cargo carriers. This work not only maps the internal landscape of SCNPs with high resolution but also provides a methodological step toward the rational design of functional nanostructures with programmable internal environments. Overall, this knowledge is central to the future programming of the particles' functions, specifically by generating protein‐like domains inside SCNP. These domains can now be understood in terms of local folds, which might eventually enable us to mimic enzyme‐like functions based on a combination of tailored dynamics and structured internal clefts for efficient substrate binding as well as release.

## Conflict of Interest

The authors declare no conflict of interest.

## Supporting information



Supporting Information

## Data Availability

The data that support the findings of this study are available from the corresponding author upon reasonable request.
