# Supplementary material for: The Core–Shell Conformational Space of Compartmentalized Single‐Chain Nanoparticles by Paramagnetic and Hyperpolarized NMR Spectroscopy
Source: Adv Sci (Weinh). 2025 Nov 30;13(9):e10909. doi: 10.1002/advs.202510909 (PMC12904050; doi:10.1002/advs.202510909)
Supplement: Supplementary file 1 — Supporting Information [file ADVS-13-e10909-s001.docx]

**The Core-Shell Conformational Space of Compartmentalized Single-Chain Nanoparticles by Paramagnetic and Hyperpolarized NMR Spectroscopy**

*Federico Faglia^1,3^, Justus F. Thümmler^2^, Christopher Pötzl^1,3^, Milan Zachrdla^1^, Ertan Turhan^1^, Dennis Kurzbach^1,*^, Wolfgang H. Binder^2,*^*

*^1^University of Vienna, Faculty of Chemistry, Institute of Biological Chemistry, Währinger Str. 38, 1090 Vienna, Austria*

*^2^Institute of Chemistry, Faculty of Natural Science II (Chemistry Physics and Mathematics), Martin Luther University Halle-Wittenberg, Von-Danckelmann-Platz 4, D-06120 Halle (Saale), Germany*

*^3^Doctoral School on Chemistry, Faculty of Chemistry, University of Vienna, Währinger Str. 38, 1090 Vienna, Austria*

*^*^corresponding authors: [dennis.kurzbach@univie.ac.at](mailto:dennis.kurzbach@univie.ac.at); [wolfgang.binder@chemie.uni-halle.de](mailto:wolfgang.binder@chemie.uni-halle.de)*

**Supporting Information**

**Synthetical Procedures**

The SCNPs were synthesized as reported previously in Reference [1]. Full characterization including NMR spectra, IR spectra, SEC traces, AFM images, and turbidimetry data can be found in reference ^[1]^

*Precursor Polymer Synthesis:*

Poly(ethylene glycol) methyl ether methacrylate (Mn=300) (4.8 mmol, 1.44 g), azidopropyl methacrylate (0.72 mmol, 121.8 mg, 113.8 µL) and trimethylsilylpropargyl methacrylate (0.48 mmol, 94.2 mg, 101.3 µL) were disolved in 1 ml dry DMF in a Schlenk tube. 1 ml of a stock solution of cyanoisopropyl dithiobenzoate (20 mM) and azobisisobutyronitirile (4 mM) was added to the solution. The resulting mixture was degassed by five freeze-pump-thaw cycles and stirred at 80°C for 3 h. The product was precipitated in cold hexane:diethyl ether (2:1) as a pink polymer. To remove the RAFT-endgroup, the polymer was solved in DMF, 10 mg of AIBN were added and the solution degassed by bubbling with N_2_. The solution was stirred at 70°C for 2 h. The product was again precipitated in cold hexane:diethyl ether (2:1). The resulting yellow polymer was purified by dialysis in THF. The highly viscous product was stored in DCM at 5°C to prevent auto crosslinking. Yield: 52%. GPC (THF): M_n_ = 36.1 kDa, M_n_/M_w_ = 1.7. ^1^H-NMR (CDCl_3_, 500 MHz, δ in ppm): 4.66-4.55 (C*H_2_*-≡-Si(CH_3_)_3_) 4.25‑3.90 (COOC*H_2_*), 3.82‑3.49 (OCH_2_C*H_2_*O), 3.48‑3.43 (N_3_C*H_2_*), 3.39 (OC*H_3_*), 2.07‑1.72 (C*H_2_*), 1.12‑0.77 (C*H_3_*), 0.20 (Si(C*H_3_*)_3_). IR (KBr): 2178 cm^-1^ (ν_alkyne_), 2100 cm^-1^ (ν_N3_). Because of peak overlapping, the found values for a’, b’ and c’ were approximated by peak integraion to be 0.83, 0.1 and 0.07

**Figure S1.** NMR Spectrum of the precursor polymer in CDCl3 (with residues of THF since it was stored as a THF solution):

*SCNP Synthesis:*

The precursor polymer (200 mg) and sodium ascorbate (300 mg, 1.5 mmol) were solved in 19 ml degassed H_2_O. A solution of TBAF x 3 H_2_O (116 µmol, 36.57 mg) and PMDETA (48 µmol, 8.3 mg, 10 µl) in 1 ml degassed H_2_O was added. The resulting solution was put into a syringe pump (1 ml/h) and added to a solution of sodium ascorbate (2.76 mmol, 574.22 mg), CuSO_4_ x 5 H_2_O (0.29 mmol, 72.37 mg) and PMDETA (0.58 mmol, 100 mg, 121 µl) in 100 ml degassed H_2_O. After 20 h the solution was stirred for one additional hour. To label the resulting SCNPs a solution of sodium ascorbate (0.5 mmol, 100 mg) in 5 ml degassed H_2_O was added to the reaction mixture. After 10 min a solution of 15 mg of the alkyne-modified TEMPO in 5 ml THF was added and the reaction mixture was stirred for 2h. The product was extracted with DCM. The DCM was evaporated under vacuum and the solid product was washed multiple times first with THF/PMDETA then with THF, and eventually with water. The product was dried under vacuum. Yield: 54%. ^1^H-NMR (D_2_O, 500 MHz, δ in ppm): 4.32‑3.90 (COOC*H_2_*), 3.82‑3.38 (OCH_2_C*H_2_*O), 3.30 (OC*H_3_*), 2.07‑1.66 (C*H_2_*), 1.14‑0.58 (C*H_3_*).

**Figure S2.** NMR Spectrum of SCNP in CDCl3.

**Figure S3.** SEC traces of the precursor Polymer and the SCNP in THF. The sample peak is completely shifted to higher retention times, proving the successful single-chain collapse and the absence of multi-chain aggregates.


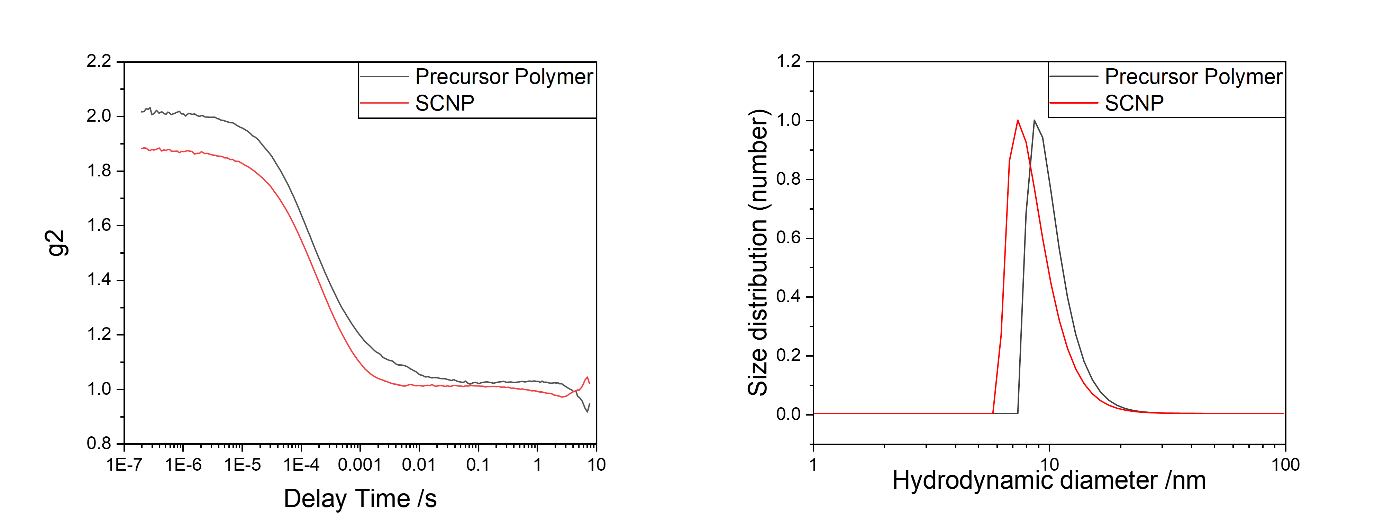


**Figure S4.** DLS Data of the Precursor Polymer and the SCNP. Left: Correlation function, right: number weighted size distribution.

**Figure S5.** Turbidimetry: T_cp, Polymer_ = 48°C, T_cp, SCNP_ = 65°C.

*Sample Preparation*

For NMR spectroscopy, the SCNPs were dissolved in pure D_2_O through stirring and sonication. Samples were first stirred in D_2_O until a turbid mixture was achieved. Then the samples were put into a sonication bath in ice water for 3 – 5 h, followed by stirring overnight. This procedure was repeated until a clear solution was achieved.

To prove that this procedure is not harming the so dissolved SCNPs, SEC measurements in THF were conducted after the complete dissolution, as well as after one and two more days of the same treatment. As visible in Figure S6 no observable shift of the peak around 7.6 min can be seen.

**Figure S6.** SEC trace of the SCNP sample in THF after multiple days of sonication. The system peaks starts at 8.8 min.The elution peak of the SCNP remains unchanged after several sonication cycles, proving that the SCNP is not degraded during the sonication prcedures.

*NMR Spectroscopy*

^1^H- one-dimensional, CPMG, NOESY, and HSQC experiments were recorded on a 600 MHz Bruker NEO spectrometer equipped with a cryogenically cooled Prodigy TCI probe. The 1D spectra with water suppression were recorded with the ‘zggw5’ pulse program of the Bruker TopSpin 4 pulse sequence catalog. The carrier frequency (O1p) was set to 4.7 ppm, and the spectral width (SW) was 24 ppm at a total acquisition time of 1.1 s. ^1^H-^1^H NOESY was recorded using the ‘noesygpph19’ pulse sequence with 512 complex *t*_1_ increments of 170 μs each (O1p = 4.7 ppm; SW = 9.8 ppm in both dimensions). The mixing time τ was 0.5 s.

Natural abundance ^1^H-^13^C HSQC was recorded using the ‘hsqcetgpsi’ pulse sequence (echo-antiecho) with 1024 complex *t*_1_ increments of 40 μs each (O1p = 4.7 ppm; SW =13.0 ppm for ^1^H and O1p = 75 ppm; SW =165.0 ppm for ^13^C).

For relaxation measurements, the CPMG (Carr-Purcell-Meiboom-Gil)-type pulse sequence ‘cpmg_espg2d’ using 17 delays (thereof two repetitions) with 0.5 ms to 1000 ms and a constant CPMG block duration of 0.5 ms (O1p = 4.7 ppm; SW = 16.0 ppm).

All data were processed using TopSpin 4 and home-written scripts for MATLAB 2022b and Python 3.12.3. Before Fourier transform, all data were baseline corrected using 5^th^-order polynomials and apodized using either exponential (for 1D) or 60° shifted squared sine bell (for 2D) functions.

The relaxation data were evaluated by binning the ^1^H spectrum into regions of 0.015 ppm in width, taking the bulk integral over each bin *I*(τ) at a given relaxation delay τ and fitting the delay-dependence of the resulting integrals to a mono-exponential decay function of the form:

*I*(τ) = *I*(0) exp(-*R*_2_τ) (1)

The PRE rate was then evaluated as the difference Δ*R*_2_ in rates found for the intact polymer and a reduced version. This later variant was obtained by treating the SCNP solution with 2 eq. sodium ascorbate for 30 min at room temperature, followed by filtration with a 1000 Da cut-off Centricon.

For the analysis of the PRE data, the spectral intensities were summed over bins of 3.06 Hz at a digital resolution of 0.18 Hz (17 data points/bin) to achieve a compromise between SNR and resolution. The narrowest feature we detected in our spectra had a linewidth of 2.9 Hz, which roughly matched the bin width. Further reductions in the bin size are therefore not expected to lead to significant changes in the reported PRE rates.

The Lorentzian fits in Fig. 2 were achieved by least-squares fitting of the line shape *L*(δ) to the following equation:

*L*(δ) = *A⋅*(Γ/2)^2^ *⋅* [(δ−δ_0_)^2^+(Γ/2)^2^]^-1^ (2)

With *A,* the signal amplitude, Γ, the linewidth, and δ, the chemical shift.

*Dissolution DNP*

For DNP, 200 μL of a solution of 15 mM TEMPOL in a mixture of 50% glycerol-d_8_, 40% D_2_O, and 10% H_2_O was hyperpolarized at 1.4 K in a magnetic field of 6.7 T for 2500 s using continuous-wave microwave irradiation at 188.08 GHz. DNP samples were always freshly prepared to avoid ripening effects.^[2]^ A VDI microwave source was used together with a 16x frequency multiplier that provided an output power for the microwave of ca. 50 mW. The magnet-cryostat combination was purchased from Cryogenic Ltd. and operated as described in reference ^[3]^.

For detection of the solid-state polarization, a 400 MHz Bruker NEO system was adapted to a ^1^H resonance frequency of 285.3 MHz and a ^13^C frequency of 71.72 MHz by using a broadband preamplifier for both channels. The detection circuit and the external tune-and-match system were home-built, as described in reference ^[4]^. To monitor the build-up, detection pulses with a flip angle of 1 degree were applied every 5 s.

After DNP, the sample was dissolved with a burst of 5 mL D_2_O at 1.5 MPa, as described in reference ^[3]^. The hyperpolarized liquid was then pushed with helium gas at 0.7 MPa to the detection spectrometer. The dissolution process employed a home-built pressure heater, actuated with an Arduino microcontroller. A home-written MATLAB-based user interface controls the dissolution and injection steps.

Detection was carried out using a 700 MHz Bruker NEO spectrometer equipped with a QCIF helium-cooled cryogenic probe. The detection pulse sequence corresponded to a series of single-pulse experiments (excite and detect). We used PC9 selective 90° covering a bandwidth of 5 ppm centered around a carrier frequency of 2 ppm. The recycling delay d1 was set to 1 s, and the additional acquisition time was 0.4 s. The hyperpolarized spectra shown in Fig. 4 correspond to the mean of the first three acquisitions. The thermal equilibrium references were recorded by repeating the same experiment on the same sample after waiting 5 min. for the hyperpolarization to completely decay.

The SCNP solutions for dDNP contained 2 mg/mL of polymer before three-fold dilution with hyperpolarized water.

Note that reference experiments with and without TEMPOL showed that no significant solvent PREs influence the spectra in the dDNP experiments. The TEMPOL concentration after dissolution and mixing was only 0.125 mM, such that no significant effects occurred.

*Molecular Dynamics Simulations*

Initial models were built using ChemDraw Office. The structures were energy minimized using MP2 molecular dynamics until the energy gradient norm was less than the minimum norm. The resulting models were fed into MD simulations performed on three different Workstation PCs. The first is equipped with one Intel® Core™ i9-12900KS processor (16c/24t), 64 GB DDR5-4800 MT/s RAM and one NVIDIA RTX 3090Ti GPU (Driver version: 515.65.01/ CUDA version: 12.1). The Operating System was Rocky Linux 9 (Kernel version: 5.14) with the GCC/G++ compiler version of 11.2.). The used GROMACS version was 2022.2.

The second is equipped with one Intel® Core™ i7-11700K processor (8c/16t), 64 GB DDR4-3200 MT/s RAM, and one NVIDIA RTX 3090 GPU (Driver version: 550.120/ CUDAversion 12.4). The Operating System was Linux Mint 22.1 (Kernel version: 6.4.8) with the GCC/G++ compiler version of 13.3.0. The used GROMACS version was 2025.2.

The third is equipped with one AMD Ryzen Threadripper PRO 5995WX processor (64c/128t), 128 GB DDR4-2666 MT/s ECC RAM, and one NVIDIA RTX 3090Ti GPU (Driver version: 555.42.06/ CUDA version 12.5). The Operating System was Linux Mint 21.2 (Kernel version: 5.15) with the GCC/G++ compiler version of 11.4. The used GROMACS version was 2024.5.

The GROMACS^[5]^ software package was used to set up and run the simulations. The used force field was the all-atom additive AMBER14SB_OL15. The molecules were parameterized using the GAFF2 force field in acpype 2023.10.27 ^[6]^ with Antechamber^[7]^ from AmberTools 24.8.

All simulations contained 1 polymer within a box of 5.5^3^ nm^3^ solvated in water using the SPC/E water model.

Energy minimization was performed using the steepest descent algorithm. The V-rescale thermostat was used for NVT equilibration for 2 ns. After NVT equilibration, NPT equilibration was performed using the C-Rescale barostat for 2 ns. The production run under NPT conditions lasted 1000 ns, with a time step of 2 fs.

Statistical Analysis

For the distance histograms presented in Fig. S11 of the Supporting Information, the statistical significance analysis was performed *via* the chi-square test. Pre-processing: the data were not normalized to yield a total probability density matching the number of analyzed atoms. No outliers were excluded. All determined distances were directly fed into the statistical analysis. Data presentation: Distance distributions. Sample size: 100 structures from each MD run. All chi-square values showed that the data was not normally distributed, proving the appearance of distinct maxima. Used software: MATLAB 2023a.

**Supplementary NMR Data**

**Table S1.** Resonance assignments.


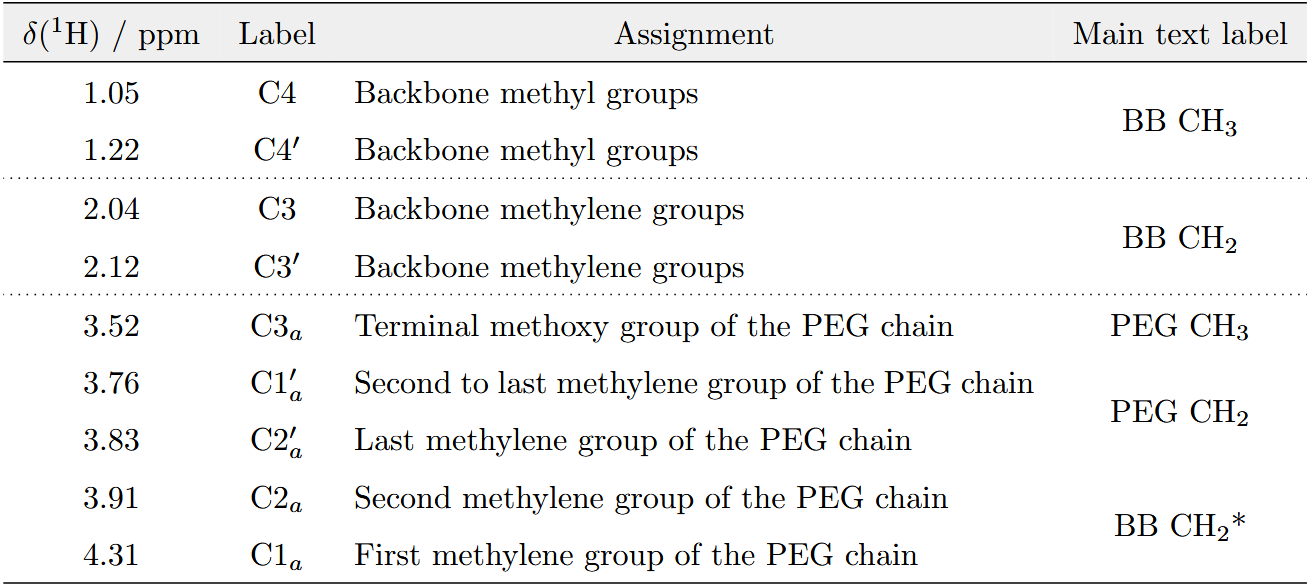


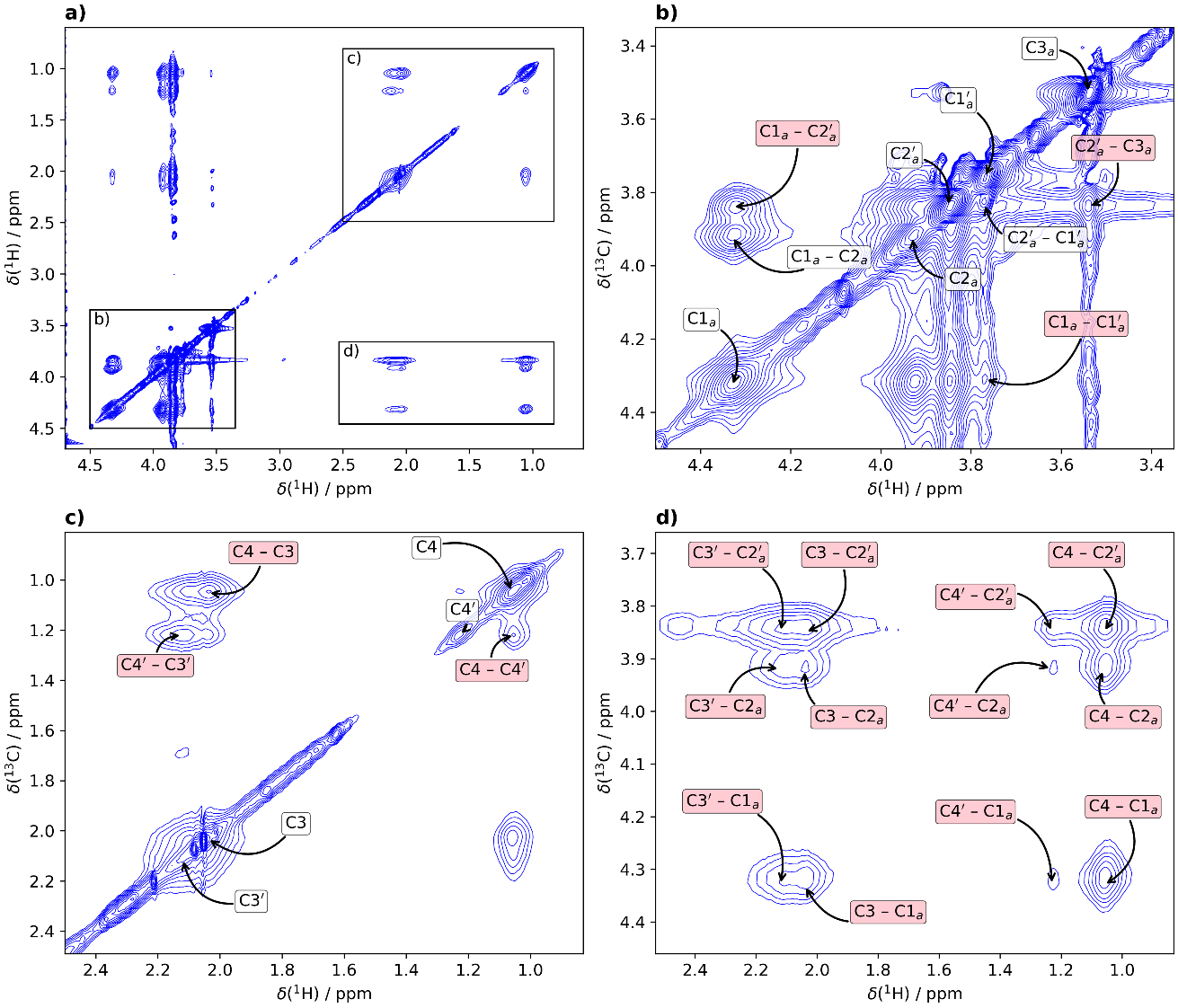


**Figure S7.** Detailed assignment of resonances in the NOESY spectrum of the TEMPO-labelled SCNP.


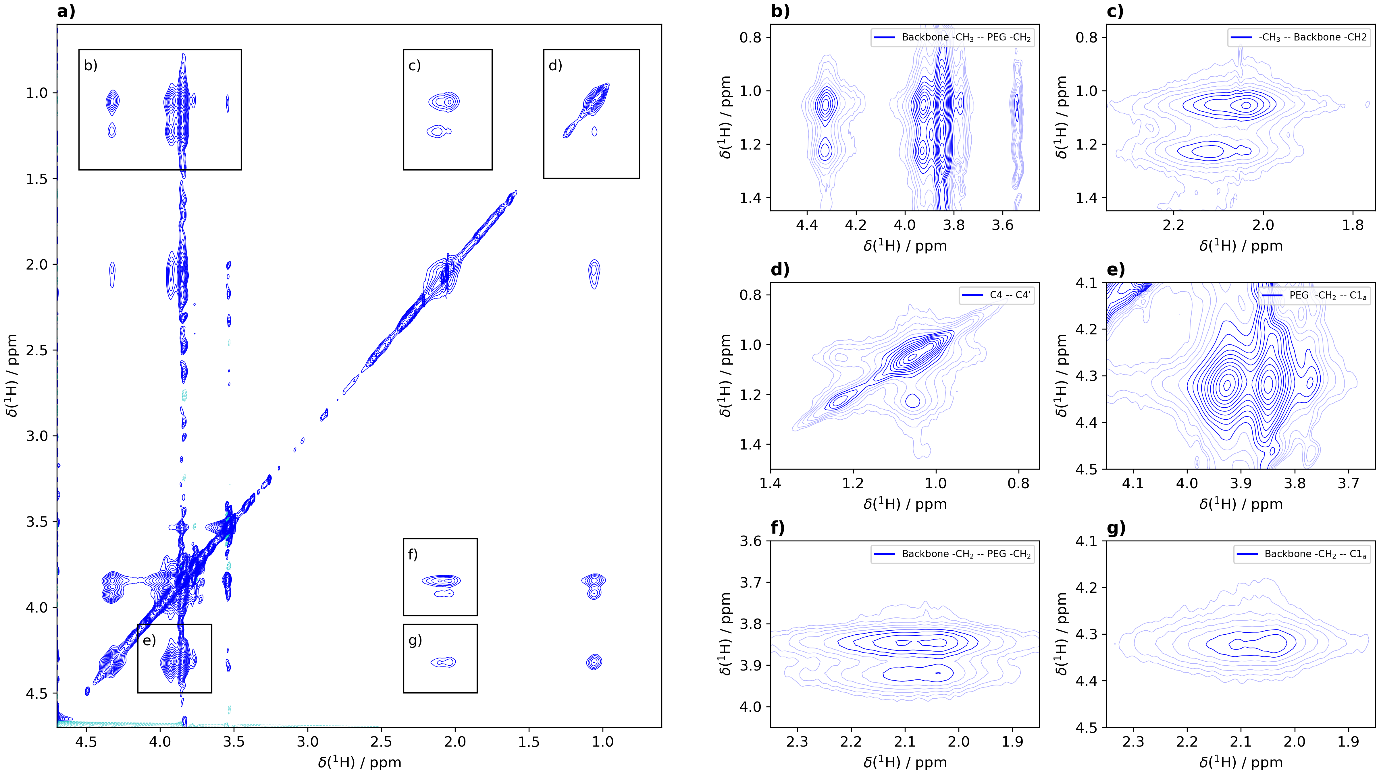


**Figure S8.** Supplementary zoom-ins on the NOESY spectrum showing peak duplication (sharp resonances superimposed on broad ones), including pegCH_3_-to-BB-CH_2_ contacts (panel b).


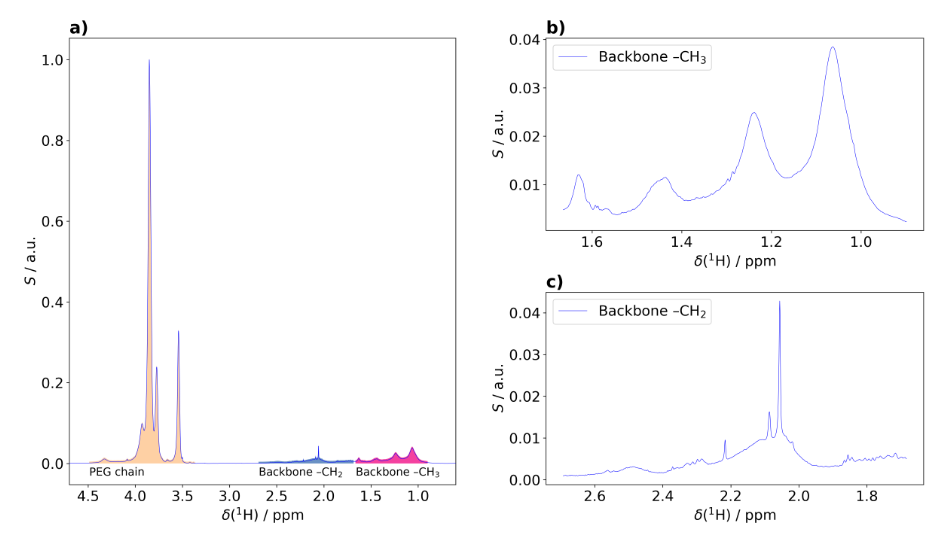


**Figure S9.** Supplementary one-dimensional ^1^H spectra again showing peak duplication (sharp resonances superimposed on broad ones), particularly well visible for the BB-CH_2_ region.


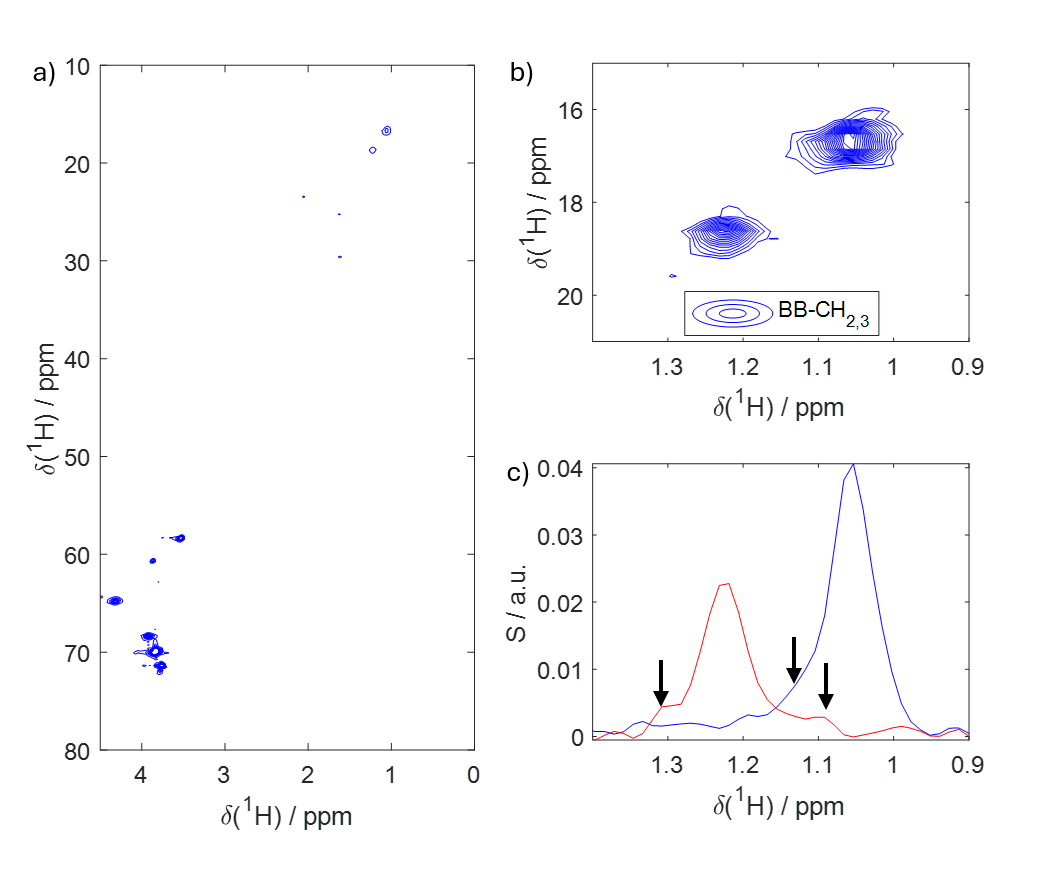


**Figure S10**. a) ^1^H-^13^C HSQC data again show the superposition of two distinct resonances for methyl and methylene groups. b) Zoom-in onto the BB methyl and methylene region. c) Sum projections onto the directly detected dimension. The black arrows indicate the broadened features.


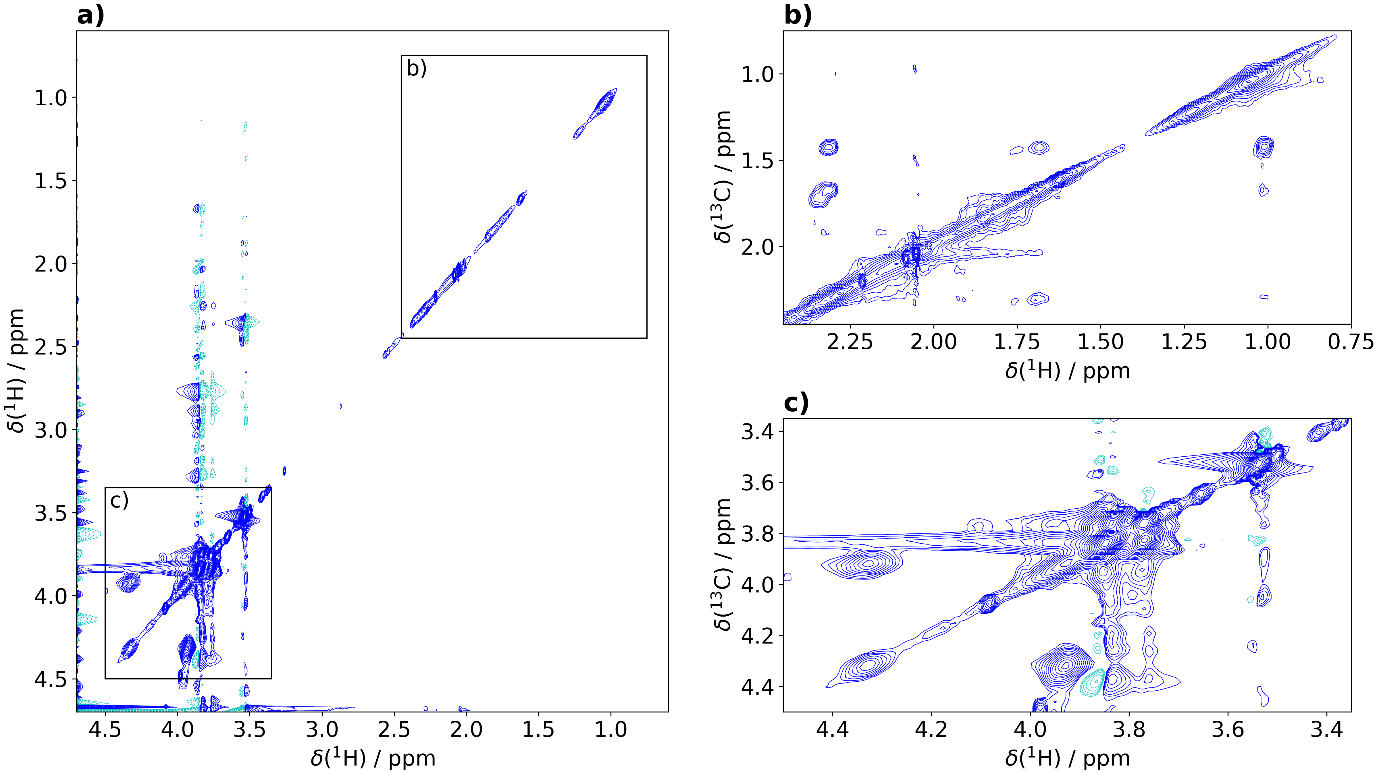


**Figure S11**. a) Supplementary ^1^H-^1^H TOCSY showing that the NOESY PEG-to-BB cross peaks are due to through-space interactions via their absence in the TOCSY. b-c) Zoom ins with different contour levels.


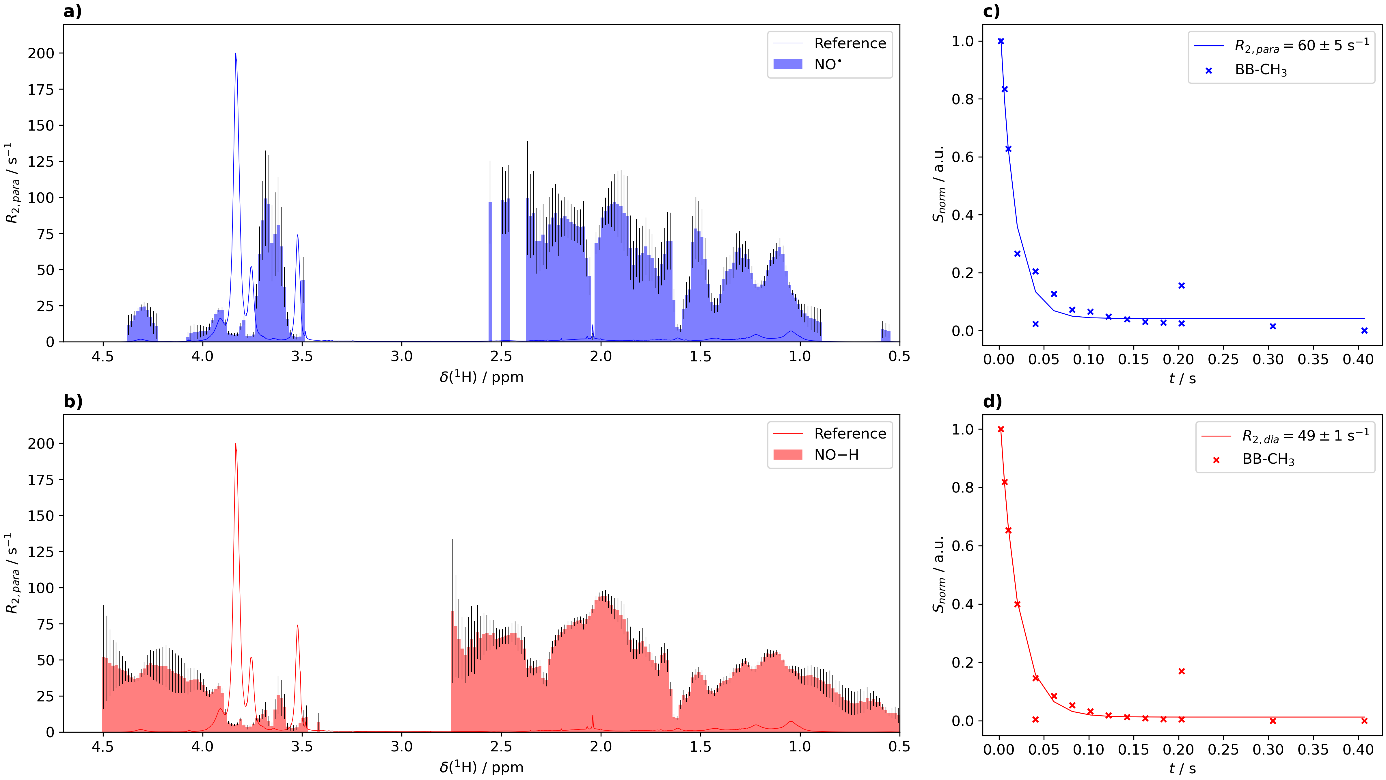


**Figure S12**. Supplementary PRE data. a) *R*_2_ rate constants with the active SL superimposed on the reference spectrum. b) *R*_2_ rate constants with the reduced SL. The differences between these rates give rise to the PRE data shown in the main text. Zero values indicate regions, where no reliable data could be evaluated due to weak signal intensities.


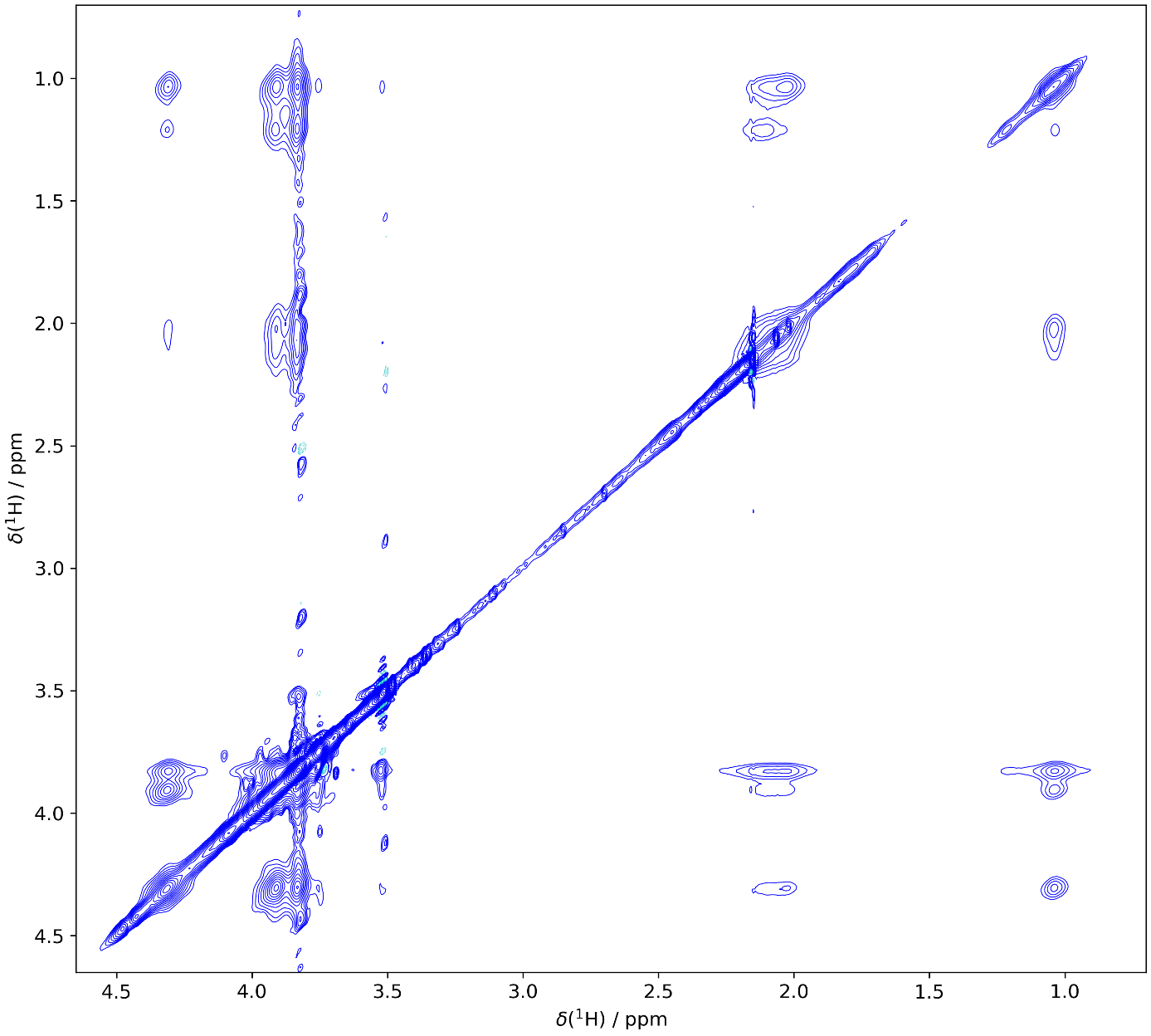


**Figure S13**. NOESY of the SCNP with TEMPO replaced by Rhodamine B. The cross peaks show a similar broad line shape as the TEMPO variant, ruling PRE effects out as a source of line broadening.


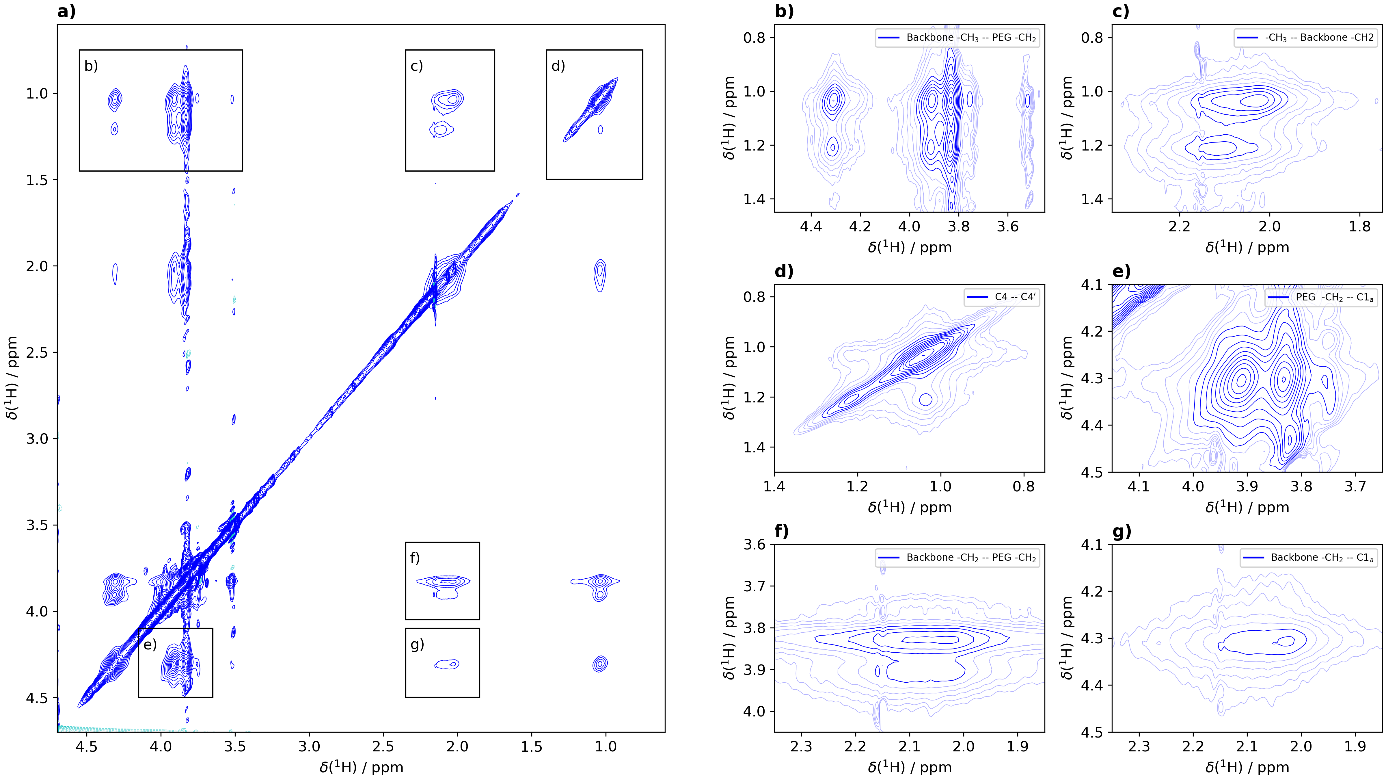


**Figure S14.** NOESY spectrum of the SCNP labelled with Rhodamine B. The full spectrum is depicted in a). Details of the backbone methyl region are depicted in d), and the cross peaks from this region to the PEG chains and the backbone methylene ones are depicted in b) and c), respectively. Cross peaks within the PEG chain spectral region are depicted in e). The cross peaks from the backbone methylene region to the PEG chains are depicted in f) and g).


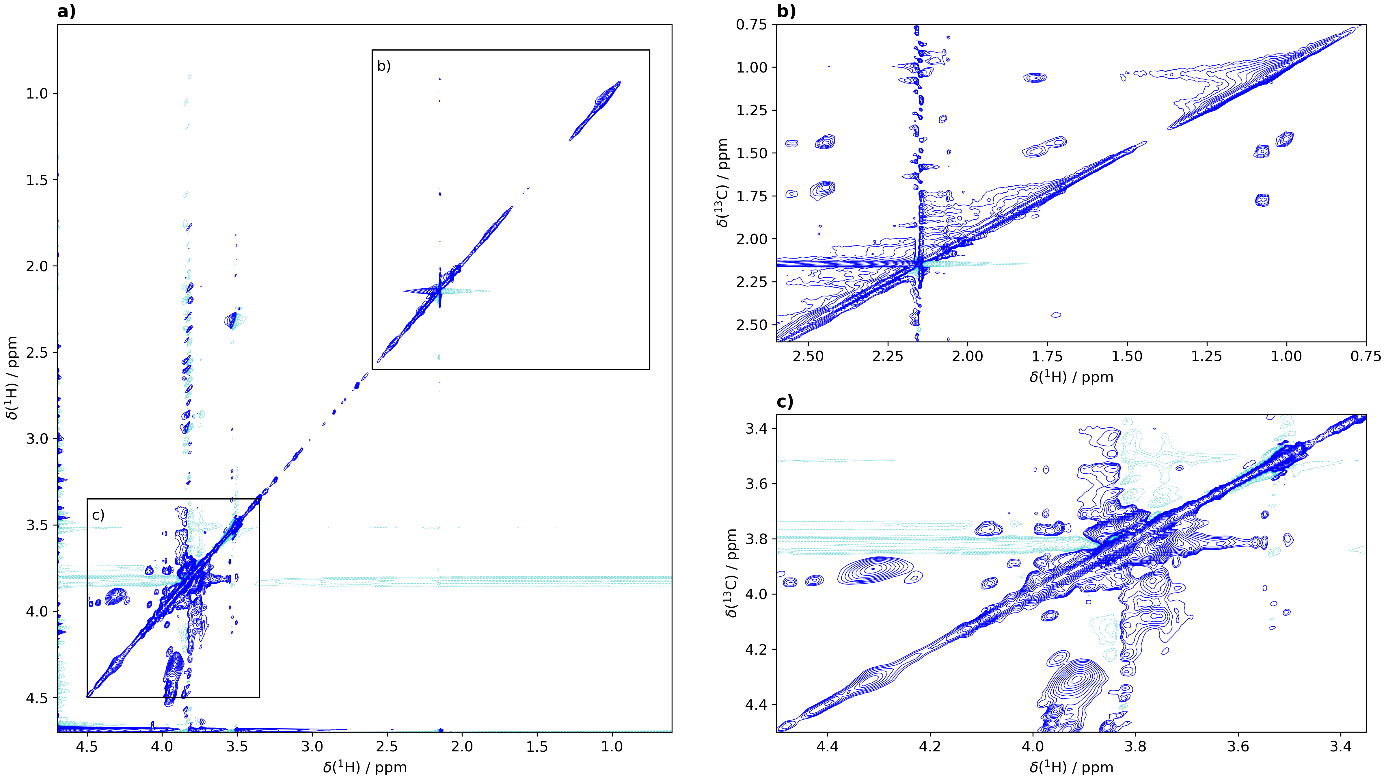


**Figure S15.** TOCSY spectrum of the SCNP labelled with Rhodamine B. The full spectrum is pictured in a); a detail of the backbone region is pictured in b); and a detail of the PEG chain region is pictured in c).


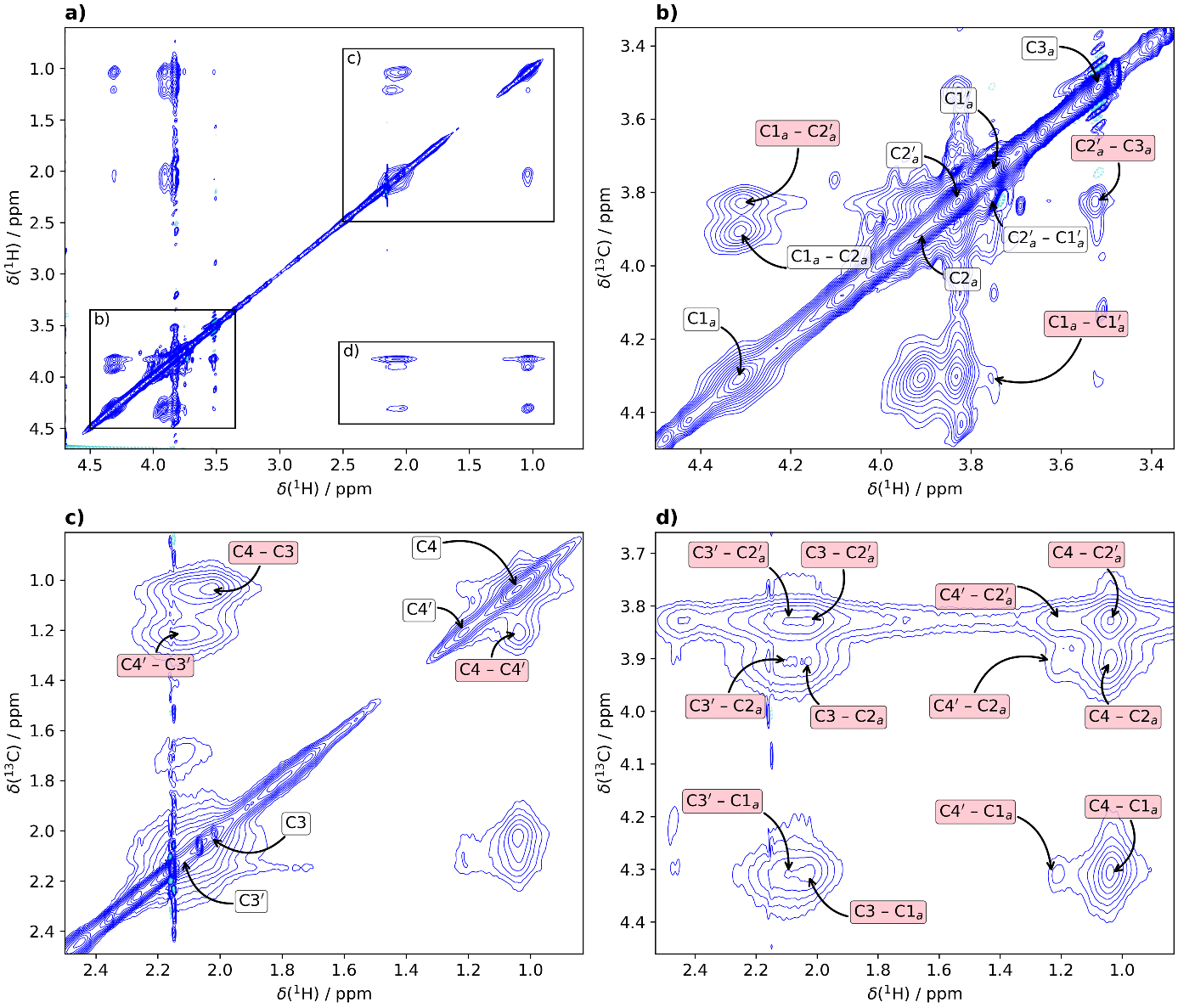


**Figure S16.** NOESY spectrum of the Rhodamine B labelled SCNP with the cross peak assignment. The cross peaks marked in pink are NOESY contacts (i.e. they don’t overlap with TOCSY cross peaks). In a) the full spectrum is depicted for reference; in b) the PEG chain region is depicted; in c) the backbone region; and in d) the cross peaks from the backbone methylene signals to the PEG chain.


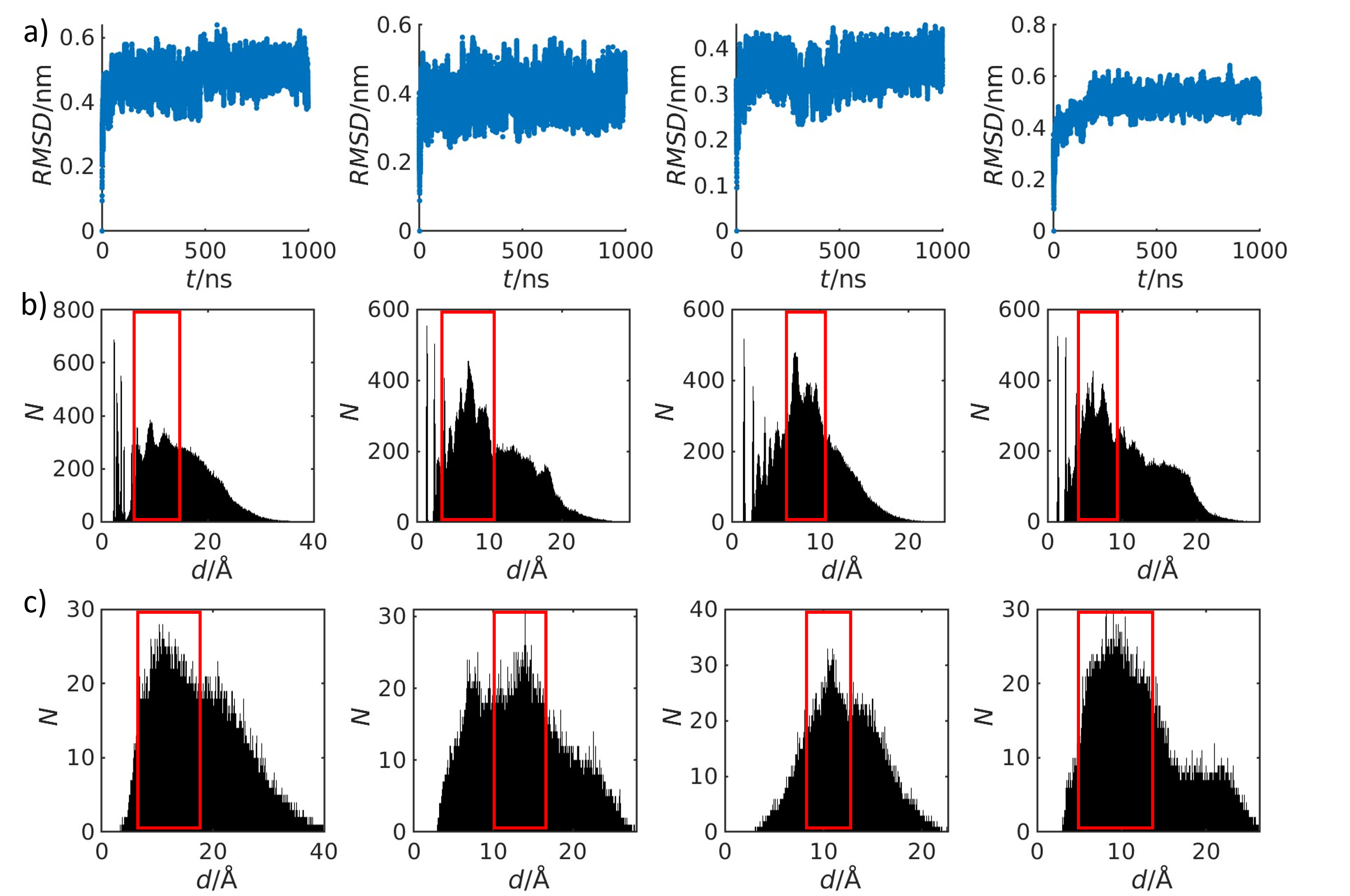


**Figure S17**. a) RMSD trajectories for the four molecular dynamics simulations of the minimal SCNP models. b) Distance histograms (nitroxide-NO to all carbon centers) for all the trajectories shown in panel (a). The appearance of distance populations with distinct maxima (marked by the red squares) clearly separated from the long distances, indicates the formation of compacted compartments distinct from the bulk polymer. c) Same as in panel b, but for NO to PEG-CH_3_. Again, distinct short-distance maxima appeared. The figure shows that the snapshots in the main text are statistically grounded. Sample sizes: 100 snapshots from the MD runs. Significance test: chi-square.


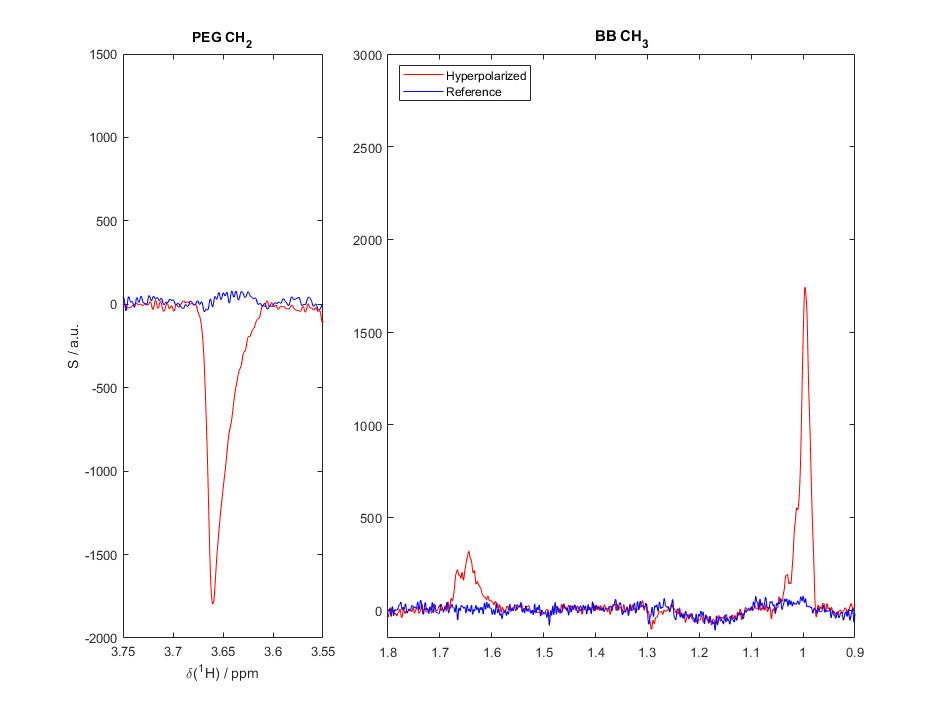


***Figure S18.*** Comparison of the hyperpolarized spectrum (red) with a conventional reference spectrum (blue) obtained with the same sample (Rhodamine B labelled SCNP) and experiment, but after decay of the hyperpolarization. While the former shows strong signal intensities for a subset of sharp resonances, the latter only led to weak signals. Notably, the PEG CH_2_ resonance is inverted under HyperW conditions. These data confirm the dDNP results shown in the main text.

*Note on the signal enhancement on the NOE sign:*

In our d-DNP experiments using HyperW, we detect the proton signals within approximately 1 s after mixing. This early detection enables the observation of direct NOE transfer from solvent to solute protons with short evolution before relayed NOEs become dominant.

Under these conditions, we observe negative NOE signal enhancements for the PEG side chains, and positive enhancements for the methacrylate backbone. This behavior reflects the well-established dependence of the NOE sign on the rotational correlation time (τ_c_​) of the interacting protons.

The NOE cross-relaxation rate σ_IS_ for two spins I and S (SCNP proton and HyperW proton in the present case) is governed by the spectral density function J(ω), and can be approximated as:

σ_IS_ ∝ J(ω_I_-ω_S_) - 6J(ω_I_) + 6J(ω_s_) + J(ω_I_+ω_S_) (1)

Under simple spherical rotation

J(ω) = τ_c_​(1+(τ_c_​ω)^2^)^-1^ (2)

In the fast-motion regime (τc≪1/ω0​), characteristic of the mobile PEG chains, this expression yields negative NOE enhancements. By contrast, the slower dynamics of the backbone, particularly within compacted compartments, push the system toward the intermediate motional regime, where the NOE becomes positive.

Thus, the observed sign inversion between PEG and backbone signals directly reflects their differential mobility. The PEG chains remain solvent-exposed and dynamically unrestricted, while the backbone is shielded within compact, conformationally restrained domains. This dynamic contrast provides independent confirmation of the core–shell morphology revealed by PRE and conventional NOESY measurements.

**References**

[1] J. F. Hoffmann, A. H. Roos, F. J. Schmitt, D. Hinderberger, W. H. Binder, *Angew. Chem. Int. Ed.* **2021**, *60*, 7820-7827.

[2] E. M. M. Weber, G. Sicoli, H. Vezin, G. Frébourg, D. Abergel, G. Bodenhausen, D. Kurzbach, *Angew. Chem. Int. Ed.* **2018**, 10.1002/anie.201800493.

[3] T. Kress, K. Che, L. M. Epasto, F. Kozak, M. Negroni, G. L. Olsen, A. Selimovic, D. Kurzbach, *Magnetic Resonance* **2021**, *2*, 387-394.

[4] M. Baudin, B. Vuichoud, A. Bornet, J. Milani, G. Bodenhausen, S. jannin, *J Magn Reson* **2018**, *294*, 115121.

[5] a) H. Bekker, H. J. C. Berendsen, E. J. Dijkstra, S. Achterop, R. Vondrumen, D. Vanderspoel, A. Sijbers, H. Keegstra, M. K. R. Renardus, in *4th International Conference on Computational Physics (PC 92)* (Eds.: R. A. DeGroot, J. Nadrchal), World Scientific Publishing, SINGAPORE, **1993**, pp. 252-256; b) H. J. C. Berendsen, D. van der Spoel, R. van Drunen, *Computer Physics Communications* **1995**, *91*, 43-56; c) E. Lindahl, B. Hess, D. van der Spoel, *Molecular modeling annual* **2001**, *7*, 306-317; d) D. Van Der Spoel, E. Lindahl, B. Hess, G. Groenhof, A. E. Mark, H. J. C. Berendsen, *Journal of Computational Chemistry* **2005**, *26*, 1701-1718; e) B. Hess, C. Kutzner, D. van der Spoel, E. Lindahl, *Journal of Chemical Theory and Computation* **2008**, *4*, 435-447; f) S. Pronk, S. Páll, R. Schulz, P. Larsson, P. Bjelkmar, R. Apostolov, M. R. Shirts, J. C. Smith, P. M. Kasson, D. van der Spoel, B. Hess, E. Lindahl, *Bioinformatics* **2013**, *29*, 845-854; g) M. J. Abraham, T. Murtola, R. Schulz, S. Páll, J. C. Smith, B. Hess, E. Lindahl, *SoftwareX* **2015**, *1-2*, 19-25; h) S. Páll, M. J. Abraham, C. Kutzner, B. Hess, E. Lindahl, in *Solving Software Challenges for Exascale* (Eds.: S. Markidis, E. Laure), Springer International Publishing, Cham, **2015**, pp. 3-27.

[6] A. W. Sousa da Silva, W. F. Vranken, *BMC Research Notes* **2012**, *5*, 367.

[7] a) J. Wang, W. Wang, P. A. Kollman, D. A. Case, *J Mol Graph Model* **2006**, *25*, 247-260; b) J. Wang, R. M. Wolf, J. W. Caldwell, P. A. Kollman, D. A. Case, *J Comput Chem* **2004**, *25*, 1157-1174.
